# Sepsis risk factors in infants with congenital diaphragmatic hernia

**DOI:** 10.1186/s13613-017-0254-9

**Published:** 2017-03-21

**Authors:** Michaël Levy, Nolwenn Le Sache, Mostafa Mokhtari, Guy Fagherazzi, Gaelle Cuzon, Benjamin Bueno, Virginie Fouquet, Alexandra Benachi, Sergio Eleni Dit Trolli, Pierre Tissieres

**Affiliations:** 1 Pediatric Intensive Care and Neonatal Medicine, Paris South University Hospitals, Assistance Publique Hôpitaux de Paris, 78, Rue du Général Leclerc, 94270 Le Kremlin-Bicêtre, France; 2 Centre de référence Maladie Rare: Hernie de Coupole Diaphragmatique, 94270 Le Kremlin-Bicêtre, France; 3 INSERM U1018, Center for Research in Epidemiology and Population Health (CESP), Paris South University, 94805 Villejuif, France; 4 Bacteriology-Hygiene Unit, Paris South University Hospitals, Assistance Publique Hôpitaux de Paris, Le Kremlin-Bicêtre, France; 5 Pediatric Surgery, Paris South University Hospitals, Assistance Publique Hôpitaux de Paris, Le Kremlin-Bicêtre, France; 6 School of Medicine, Paris South University, UPS11, Le Kremlin-Bicêtre, France; 7 Obstetrics, Gynecology and Reproductive Medicine, Antoine Béclère Hospital, Assistance Publique Hôpitaux de Paris, Clamart, France; 8 Institute of Integrative Biology of the Cell, CNRS, CEA, Univ. Paris Sud, Paris Saclay University, Gif-sur-Yvette, France

**Keywords:** Congenital diaphragmatic hernia, Sepsis, Healthcare-associated infection, Ventilator-associated pneumonia, Central line-associated bloodstream infection

## Abstract

**Background:**

Congenital diaphragmatic hernia (CDH) is a rare congenital anomaly and remains among the most challenging ICU-managed disease. Beside severe pulmonary hypertension, lung hypoplasia and major abdominal surgery, infective complications remain major determinants of outcome. However, the specific incidence of sepsis as well as associated risk factors is unknown.

**Methods:**

This prospective, 4-year observational study took place in the pediatric intensive care and neonatal medicine department of the Paris South University Hospitals (Le Kremlin-Bicêtre, France), CDH national referral center and involved 62 neonates with CDH.

**Main results:**

During their ICU stay, 28 patients (45%) developed 38 sepsis episodes. Ventilator-associated pneumonia (VAP: 23/38; 31.9 VAP per 1000 days of mechanical ventilation) and central line-associated blood stream infections (CLABSI: 5/38; 5.5 per 1000 line days) were the most frequently encountered infections. Multivariate analysis showed that gestational age at birth and intra-thoracic position of liver were significantly associated with the occurrence of sepsis. Infected patients had longer duration of mechanical and noninvasive ventilation (16.2 and 5.8 days, respectively), longer delay to first feeding (1.2 days) and a longer length of stay in ICU (23 days), but there was no difference in mortality.

**Conclusions:**

Healthcare-associated infections, and more specifically VAP, are the main infective threat in children with CDH. Sepsis has a significant impact on the duration of ventilator support and ICU length of stay but does not impact mortality. Low gestational age and intra-thoracic localization of the liver are two independent risk factors associated with sepsis.

**Electronic supplementary material:**

The online version of this article (doi:10.1186/s13613-017-0254-9) contains supplementary material, which is available to authorized users.

## Background

Congenital diaphragmatic hernia (CDH) is a rare congenital anomaly of the diaphragm with an incidence of approximately 1–3.5 per 10,000 births [1–3]. CDH patients suffer from severe respiratory failure caused by a combination of defect in the muscular or tendinous portion of the diaphragm, pulmonary hypoplasia and persistent pulmonary hypertension (PAH) [4]. Once the diaphragm is surgically repaired, lung morbidities are considered to be the main determinant for postnatal outcome, resulting in an overall survival rate up to 84% [5]. With the improvement in neonatal care of children with CDH, other factors than pulmonary hypoplasia and PAH impact on the prognosis like nutrition and infections [6, 7]. It is recognized that following the initial neonatal period, recurrent respiratory infections are frequently reported complications and are responsible for most hospital readmissions [7]. However, there are very few data concerning sepsis and healthcare-associated infection (HCAI) in this population and its impact on prognosis.

The aim of this study is to evaluate the incidence of sepsis and HCAI in children with CDH and to identify the associated risk factors.

## Methods

We performed prospective, monocentric study that included all consecutive neonates born with a CDH who were between December 2009 and January 2015 in the neonatal intensive care unit of the Paris South University Hospitals in Kremlin-Bicêtre, France. Our center is a national referral center for CDH management. During the study period, 161 pregnant mothers with prenatally diagnosed CDH were referred from other centers for antenatal evaluation for potential inclusion in fetal tracheal occlusion (FETO) trials (AB; TOTAL Trial 1 NCT007637737; TOTAL Trial 2 NCT01240057). Subsequently, 82 (51%) with the most severe CDH form were selected and oriented to our center for perinatal management. These patients were identified from a prospectively maintained database of all newborn admitted in our unit with congenital diaphragmatic hernia. In addition, patients with sepsis were identified in a prospective HCAI and sepsis surveillance program and matched with the institutional program of medicalization of information systems (PMIS) database using the 10th version of the International Classification of Diseases. All newborns dying within the first 3 days of life were excluded. Additional data missing in the prospective database were retrospectively collected from patient’s files. All patients’ data were secondarily anonymized. Children representatives were informed. The study was reviewed by the Ethic Committee of the French Society of Intensive Care (Société de Reanimation de Langue Française), which waived the need of parents signed informed consent (CE SRLF-15-38).

The primary endpoint was to describe the incidence of sepsis in children with CDH. Secondary endpoints were the identification of associated risk factors and to evaluate the impact on patients’ outcome. The following data were collected for all patients: sex, gestational age at birth, gestational age at diagnosis, birth weight, hernia type (right or left), best observed/expected lung–head ratio (O/E LHR), intra-thoracic position of liver, the period of diagnosis (ante versus postnatal), fetal tracheal occlusion (FETO) during pregnancy, antenatal steroid therapy, delay between delivery and surgery, presence of surgical abdominal plate or chest tube, use of various catheters (umbilical venous and artery catheters, peripheral arterial catheter, centrally inserted central venous catheter (CVC) and peripherally inserted central catheter (PICC). Sepsis was defined as clinical symptoms of infection and/or sudden clinical deterioration together with significant increase in C-reactive protein (CRP) and/or procalcitonin (PCT). Healthcare-associated infections were defined according to the 2016 update of Center for Disease Control HCAI criteria (Additional file 1: Table S1) [8, 9].

Data on infection were also collected: type of infection, presence of septic shock, identification of bacteria, temperature, CRP, PCT, leukocyte and neutrophil counts, initial antibiotic therapy and duration, time to first negative PCT. The outcome data were durations of mechanical ventilation, duration of high-frequency oscillatory ventilation (HFOV), duration of noninvasive ventilation, time to first day of feeding, weight at day five, presence of PAH, use of pulmonary vasodilators, use of inotrope treatments, length of hospitalization stay and death.

The results of the descriptive analysis were expressed as numbers and percentages for qualitative variables and as mean and standard deviation for quantitative variables. Two-tailed Fisher’s exact test for quantitative variables and Chi-square test for qualitative variable were used. Risk of infection was modeled using a multivariate logistical regression analysis. A stepwise selection was used, and the final model was adjusted on all variables associated with a *p* value below 0.2.

## Results

### Patients characteristics

Eighty-two children were hospitalized during the study period. Sixteen children died within the first 3 days of life (38% of right CDH, no sepsis), and two additional patients were excluded because the diagnosis was made after the neonatal period (2.4 and 6 months after birth). The 62 remaining newborns (all inborn) were included in the study. Patients’ characteristics are described in Table 1. The mean gestational age at birth was 38.4 ± 2.2 weeks and the mean birth weight was 3072 ± 671 g. Ten patients (16%) were preterm. Most patients had left-sided CDH (90%, *n* = 56) and 38.7% of the patients had an intra-thoracic ascension of liver (*n* = 24). 83.9% of the patients (*n* = 52) were diagnosed during pregnancy, the mean age of CDH diagnosis was 27.6 ± 7.4 weeks and the mean of O/E LHR was 42.1%. Seven patients (11.3%) underwent a FETO procedure during pregnancy and nine (14.5%) had antenatal steroids. The mean length of hospitalization was 21.9 ± 27.8 days.

**Table 1:** Patients characteristics

	No sepsis (*n* = 34)	Sepsis (*n* = 28)	*p* value*
Gender (male)	26 (76.5%)	19 (67.9%)	0.57
Gestational age at birth (weeks)	38.8 (2.1)	37.8 (2.2)	0.09
Term of birth			
Preterm (<37 weeks)	4 (11.8%)	6 (21.4%)	0.49
Term (≥37 weeks)	30 (88.2%)	22 (78.6%)	0.79
≥37 and <39 weeks	8	11	
≥39 and <42 weeks	22	11	
Birth weight (g)	3092 (633)	3048 (726)	
Gestational age at diagnosis (weeks)	30.0 (7.5)	25.1 (6.3)	<0.01
Type			
Left	31 (91.2%)	25 (89.3%)	0.80
Right	3 (8.8%)	3 (10.7%)	
Best observed/expected LHR (%)	47.2 ± 12.2	37.8 ± 12.5	0.02
Intra-thoracic liver	7 (21.2%)*^1^	17 (60%)	<0.01
Antenatal diagnosis	26 (76.5%)	26 (92.8%)	0.08
FETO	3 (8.8%)	4 (14.3%)	0.49
Antenatal steroids	1 (2.9%)	8 (28.6%)	<0.01
Time before surgery (days)	2.26 (1.9)	2.14 (1.0)	0.74
Patch repair	4 (12.5%)*^2^	13 (46.4%)	<0.01
Chest tube	7 (25.9%)*^2^	4 (12.5%)*^1^	0.18
Umbilical venous catheter	29 (85.3%)	26 (92.9%)	0.34
Umbilical arterial catheter	4 (11.8%)	3 (10.7%)	0.89
Arterial catheter	2 (5.8%)	5 (17.9%)	0.13
Central venous catheter	17 (50.0%)	26 (92.9%)	<0.01
Peripherally inserted central venous catheter	9 (26.5%)	10 (35.7%)	0.43

Values are expressed as number (%), or mean ± SD

*^X^ *X* number of missing data; *LHR* lung-to-head ratio; *FETO* fetal tracheal occlusion

* Univariate analysis

### Description of sepsis episodes

Twenty-eight patients (45%) developed 38 sepsis episodes during the hospitalization period. Four patients developed two different sepsis episodes, one patient developed three and one patient developed five episodes during the hospitalization. Clinical and laboratory findings are presented in Table 2. VAP was the main cause of sepsis and accounted for 60.5% of all cases (23/38) with an incidence density of 31.9 per 1000 days of mechanic ventilation. VAP was followed by CLABSI (5/38, 5.5 per 1000 line days, 13.2% of the infections) and by urinary tract infections and bacteremia with no source identified. Forty-seven centrally inserted CVC were used in 43 patients including 31 left subclavian vein, 15 internal jugular veins and one femoral vein CVC. CLABSI occurred in 3/47 (6.4%) centrally inserted CVC and in 2/19 (10.5%) PICC. Sepsis occurred after a mean of 22.4 ± 33.1 days of hospitalization. The mean time to develop VAP was 25.5 ± 36.1 days, and CLABSI was 35.6 ± 41.9 days. Most patients (65.8%) had fever, while 15.8% had hypothermia at diagnosis. Mean CRP value was 103.2 ± 73.5 mg/L, and mean PCT value was 10.5 ± 21.2 μg/L. The main bacteria identified were *Escherichia coli* (22%), *Staphylococcus epidermidis* (19.5%), *Staphylococcus aureus* (14.6%) and *Enterobacter cloacae* (9.8%). Vancomycin, piperacillin–tazobactam, cefotaxime, gentamicin and amikacin were the main antibiotics used following unit protocoled antibiotic therapy. Antibiotics were adapted to the bacteria and its resistance profile in all cases. Bacteria were identified in 89% of all sepsis. The mean time to normal PCT was 6.5 ± 3.4 days, and the mean duration of antibiotic therapy was 8.02 ± 2.41 days.

**Table 2:** Sepsis characterization

Data	*N* (%) or mean (SD)
Number of sepsis episodes*	38
Type of sepsis	
Meningitis	1 (2.6%)
Ventilator-associated pneumonia	23 (60.5%)
Same side as hernia	6 (26%)
Opposite side as hernia	11 (48%)
Both lungs	6 (26%)
Urinary tract infection	3 (7.9%)
With urinary catheter	1 (2.6%)
Without urinary catheter	2 (5.3%)
Central line-associated bloodstream infection	5 (13.2%)
Central venous catheter	3 (7.9%)
Peripherally inserted central catheter	2 (5.3%)
Bacteremia with no origin found	3 (7.9%)
Early-onset neonatal sepsis	2 (5.3%)
Surgical site infection	1 (2.6%)
Septic shock	7 (18.4%)
Delay between delivery and sepsis (days)	
All infections	22.4 (33.1)
VAP	25.5 (36.1)

*N* number, *SD* standard deviation, *VAP* ventilator-associated pneumonia

* In 28 patients

### Risk factors for infection

Using univariate comparison (Table 1), infected patients had a significant lower gestational age at diagnosis, lower observed/expected LHR than non-infected patients (O/E LHR range 12.5–65 vs. 27.1–69, respectively) and more use of antenatal steroids. Intra-thoracic liver, use of surgical plate and CVC were also more frequent in infected patients. The difference between the two groups regarding gender, gestational age at birth, proportion of preterm, birth weight, type of hernia, FETO, delay between delivery and surgery, requirement of a chest tube, umbilical venous and arterial catheters, peripheral arterial catheter and PICCs was not significant. After multivariate logistical regression analysis and a stepwise selection of variables (Table 3), the gestational age at birth (in weeks), the birth weight (in grams), an intra-thoracic position of liver and the presence of a centrally inserted CVC were significantly associated with the occurrence of an infection. With every additional week of gestation at birth, the OR of contracting an infection was 0.439 (95% CI 0.224–0.862). Although most children with sepsis had a centrally inserted CVC (26/28), outlining the severity of those patients, very few developed a CLABSI.

**Table 3:** Multivariate analysis of risk factor for sepsis

	Odds ratio	Wald-type 95% CI	*p* value
Gestational age at birth (weeks)	0.439	0.224–0.862	0.016
Birth weight (grams)	1.003	1.001–1.006	0.012
Right CDH	0.894	0.783–1.022	0.099
Intra-thoracic liver	8.319	1.439–48.104	0.018
Centrally inserted venous catheter	34.582	2.864–417.635	0.005
Peripherally inserted central catheter	3.836	0.627–23,485	0.145

### Impact of sepsis on patients’ outcome

Infected patients had significantly poorer prognosis than non-infected patients (Table 4). Infected patients had longer duration of mechanical ventilation, longer duration of noninvasive ventilation, longer delay to first feeding and longer duration of hospitalization. Infected patients also requested significantly more inotrope treatment, fluid resuscitation and red blood cells transfusion (data not shown). There was no significant difference in death rate in the two groups.

**Table 4:** Impact of sepsis on patients’ outcomes

	Sepsis (*n* = 28)	No sepsis (*n* = 34)	*p* value or OR (95% CI)
Duration of mechanical ventilation (days)	20.5 (15.6)	4.3 (4.0)	<0.0001
Duration of HFOV (days)	10.2 (9.9)	1.2 (2.4)	<0.0001
Duration of noninvasive ventilation (days)	6.9 (12.3)	1.1 (2.4)	<0.01
Time to first day of feeding (days)	6.0 (2.3)	4.8 (2.0)	0.049
Weight at day 5 (g)	3246 (769)	3076 (644)	0.37
Duration of hospitalization (days)	34.6 (35.9)	11.6 (11.6)	<0.001
Volemic expansion*	21 (75.0%)	14 (41.1%)	4.2 (1.4–12.8)
Inotrope treatment*	24 (85.7%)	17 (50.0%)	5.9 (1.7–21.0)
NO treatment*	20 (71.4%)	9 (26.4%)	6.9 (2.2–21.2)
PAH*	27 (96.4%)	27 (79.4%)	7.0 (0.8–60.0)
Death*	7 (25.0%)	5 (14.7%)	1.9 (0.6–6.9)

Values are expressed as mean (SD) or * number (percent)

*OR* odd ratio, *HFOV* high-frequency oscillatory ventilation, *NO* nitric oxide, *PAH* pulmonary arterial hypertension defined as tricuspid regurgitation >3 m/s associated with right ventricle and septal signs of PAH

## Discussion

The major finding of this study is that lower gestational age at birth is independently associated with sepsis and HCAI in children with CDH, representing a major risk factor for associated morbidities. Similarly, following neonatal congenital heart surgery, HCAI is known to be associated with prolonged length of stay in ICU as well as increased mortality and costs [10–14].

Although HCAI is representing the vast majority of sepsis (30/38), VAP is clearly emerging as the main cause of infection in this subset of neonatal patients. The prevalence of VAP in this population (31.9 per 1000 ventilator days) is higher than in other neonates admitted in our unit during the same period of time (2009–2014) with a mean of 16.5 per 1000 ventilator days (yearly range 9.1–19.3 per 1000). In most studies, rate of VAP varies between 0.2 and 10.9 per 1000 ventilator days, and a recent meta-analysis found a prevalence ranging from 8.1 to 57.1% [9, 15–17]. Beside local prevention bundles application, the mechanism by which neonates with CDH have higher rates of VAP is not clear. A susceptibility to pulmonary infection could be explained by lung hypoplasia including fewer alveoli, reduced vascular bed and a certain degree of reduced ciliary epithelium. The fact that the most severe cases (with liver up) were more prone to infection is in favor of this hypothesis. Hypoplastic lung immunity might not be as efficient as in normal lungs, explaining why children with CDH have an increased sensitivity to the development of VAP. Some indirect observations are supporting this hypothesis. It was shown that adult patients with pulmonary fibrosis have increased bacterial burden due to poorer lung vascularization [18]. Although altered immunity has not been described in neonates with CDH, recently, it was shown that peak inspiratory pressure impacts sphingomyelin degradation in CDH patients outlining the effects of ventilation on lung cell apoptosis and homeostasis [19]. Furthermore, role of mechanical ventilation in potentializing the innate immune response to bacteria and subsequent development of VAP is now well validated [20]. In neonates with CDH and hypoplastic lung, mechanical ventilation is associated with a high risk of barotrauma potentially aggravating secondary bacterial insults on lung parenchyma.

In our CDH cohort, we observed a microbiological shift in VAP pathogen. It is usually considered that in neonatal VAP, the most prevalent monomicrobial pathogens are *P. aeruginosa*, *K. pneumonia* and *S. aureus*, whereas polymicrobial VAP accounted for 6–58% of the cases [9]. In our series, few polymicrobial infections (3/38 episodes) were observed, and we did not found any infection due to *P. aeruginosa* but a high proportion of other Gram-negative bacteria, including *E. coli* and *E. cloacae*. In our patients, CLABSI is the second cause of sepsis. It is difficult to compare CLABSI incidence rate (5.5 par 1000 line days) to the neonatal literature (ranging between 1.43 and 11 per 1000 line days), considering that most studies include neonates from all terms (including very low birth weights) and that most neonatal units are using preferentially PICC and not centrally inserted CVC as routine practice [21–25]. Nevertheless, neonatal CLABSI incidence rates are mainly caused by *S. epidermidis* and *S. aureus* [26]. In our series, although Gram-negative sepsis burden was high (55.2%), there was no CLABSI caused by enterobacteria.

We found that children with older gestational age at delivery have reduced risk of developing an infection in the course of hospitalization. This result leads to the question of the best timing of delivery of neonates with CDH. The CDH Study Group reported increased survival among prenatally diagnosed CDH infants born at early-term ages (37–38 weeks of gestation), compared with infants born at later term ages (39–41 weeks of gestation) hypothesizing an increasing severity of lung hypoplasia with advancing gestational age [27]. However, the results have been challenged, and other studies found that neonates with CDH seemed to have inferior neonatal and infant mortality with the increase in the gestational age of birth [28–31]. Although most patients are less than 39 and more than 37 weeks of gestational age, our results clearly outline the impact of lower gestational age at delivery as a major perioperative risk factor for infective complications. The intra-thoracic position of liver is also an independent risk factor for infection in our population. Infants with intra-thoracic liver are already known to have poorer neonatal outcome with greater death before discharge and increased risk of extracorporeal membrane oxygenation (ECMO) [32–35]. The importance of liver positioning is crucial for the outcome of patients, and its link to poorer long-term outcome has been well established [32, 36]. Among the 17 infected patients with liver ascension, 12 developed a VAP adding to the probable hypothesis of a specific lung cause to the development of VAP. Controlateral pneumonia, in the so-called healthy lung, reinforces the barotrauma hypothesis and subsequent pathogenesis of VAP and raised the difficult question of optimal pressure/volume target ventilation [20, 37, 38].

Despite its monocentric design, our cohort is characterized by a highly selected case load. Interestingly, mortality (13/62; 21%) in our cohort is corresponding to those of the CDH Euro consortium (25%) as recently shown, outlining the adequacy of our results with larger European CDH centers [38]. Although one can argue that most infections were HCAI, and presumably preventable, our study suggest that neonates managed for CDH have an increased risk of developing sepsis and more specifically VAP compared to the general neonatal ICU patients. During the same period of time, in our unit, VAP incidence in CDH patients (23/62, 37.4%) was much higher than in the rest of patients ventilated for >12 h (68/715, 9.5%). Furthermore, the high incidence of VAP despite local VAP prevention bundles should raise the possibility that children with the most severe form of CDH have an intrinsic susceptibility to VAP. This has to be confirmed in multicenter cohort.

## Conclusion

This study shows that neonates hospitalized for CDH have a high incidence of sepsis, mostly related to the development of VAP. We showed that the gestational age independently affects the risk of sepsis development. However, although the impact of sepsis on hospital morbidities is strong, sepsis has no effect on mortality in neonates with CDH. This study urge confirmation and immunologic investigations aimed at studying susceptibility to infections of infants with CDH and particularly the physiopathologic basis of VAP in hypoplastic neonatal lung.

## Additional file

**Additional file 1.** Diagnostic criteria for health care associated infections (HCAI).

## Abbreviations

CDH: congenital diaphragmatic hernia
CLABSI: central line-associated bloodstream infections
CSF: cerebrospinal fluid
CVC: central venous catheter
FETO: fetal tracheal occlusion
HCAI: healthcare-associated infection
HFOV: high-frequency oscillatory ventilation
NO: nitric oxide
O/E LHR: observed/expected lung–head ratio
PICC: peripherally inserted central catheter
PAH: pulmonary arterial hypertension
VAP: ventilator-associated pneumonia
