# Supplementary material for: Sepsis risk factors in infants with congenital diaphragmatic hernia
Source: Ann Intensive Care. 2017 Mar 21;7:32. doi: 10.1186/s13613-017-0254-9 (PMC5359267; doi:10.1186/s13613-017-0254-9)
Supplement: Supplementary file 1 — Additional file 1. Diagnostic criteria for health care associated infections (HCAI). [file 13613_2017_254_MOESM1_ESM.doc]

| **SUPPLEMENTAL DIGITAL CONTENT**  **Table S1.** Diagnostic criteria for health care associated infections (HCAI)* | |
| --- | --- |
| **Ventilator Associated Pneumonia (VAP)** | |
| Radiological signs | Patient with one or more (in patients with underlying diseases two or more) chest X-rays with one of the following findings:  – new or progressive and persistent infiltrate  – consolidation  – cavitation  – pneumatoceles |
| + Clinical signs and symptoms † | Worsening of gas exchange [e.g. oxygen desaturations (e.g. pulse oxymetry < 94%), increased oxygen requirements, or increased ventilation demand] and three of the following:  – temperature instability with no other recognized cause  – leukopenia (< 4,000 WBC/mm ^3^) or leucocytosis (>15,000 WBC/mm ^3^) and left shift (> 10% band forms)  – new onset of purulent sputum, or change in the character of sputum, or increase in respiratory secretions, or increased suctioning requirements  – apnea, tachypnea, nasal flaring with retraction of chest wall or grunting  – wheezing, rales, or rhonchi  – cough  – bradycardia (< 100 beats/min) or tachycardia (> 170 beats/min) |
| + Microbiolocical  findings | At least one of the following:  – positive growth in blood culture not related to another source of infection  – positive growth pleural fluid culture  – positive quantitative culture from a minimally contaminated LRT specimen [e.g. BAL (≥ 10 ^4^ CFU/ml) or protected specimen brushing (≥ 10 ^3^ CFU/ml)]  – ≥ 5% BAL-obtained cells contain intracellular bacteria on direct microscopic examination (e.g. Gram stain)  – histopathological exam shows at least one of the following criteria for pneumonia:  abscess formation or foci of consolidation with intense PMN accumulation in bronchioles and alveoli,  positive quantitative culture of lung parenchyma (≥ 10 ^4^ CFU/g tissue), or evidence of lung parenchyma invasion by fungal hyphae or pseudohyphae |
| **Central Line-Associated Bloodstream Infection (CLABSI)** | |
| Microbiological finding | Laboratory-confirmed bloodstream infection not related to an infection at another site and where central line or umbilical catheter was in place for > 2 calendar days on the date of event and where the line was also in place on the date of event or the day before |
| **Urinary tract infection** | |
| Clinical signs and  Symptoms † | Presence of clinical signs of infection:  – temperature instability with no other recognized cause  – leukopenia (< 4,000 WBC/mm ^3^) or leucocytosis (> 15,000 WBC/mm ^3^) and left shift (> 10% band forms)  – bradycardia (< 100 beats/min) or tachycardia (> 170 beats/min) |
| + Microbiological  findings | Positive urine culture with no more than two species of organisms identified, at least one of which is a bacterium of ≥ 10 CFU/ml |
| * ABSI=ological finding eumonia ( 1 yearpos avec ording to the ée et nouveau-né ex0000000000000000000000000000000000000000000000* 2016 update Center of Disease Control criteria [8]; † adapted for infant <1 year [9].  HCAI = health care associated infections; VAP = Ventilator associated pneumonia; CLABSI = Central Line-Associated Bloodstream Infections; WBC = White blood cells; LRT = lower respiratory tract; BAL= Broncho-alveolar lavage; CFU = colony-forming units. | |
